# Human and domesticated animal environmental DNA as bioassays of the Anthropocene

**DOI:** 10.1016/j.xinn.2022.100356

**Published:** 2022-11-25

**Authors:** David S. Thaler, Jesse H. Ausubel, Mark Y. Stoeckle

**Affiliations:** 1 Biozentrum, University of Basel, CH-4056 Basel, Switzerland; 2 Program for the Human Environment, Rockefeller University, New York, NY 10065, USA

**Figure 1:**
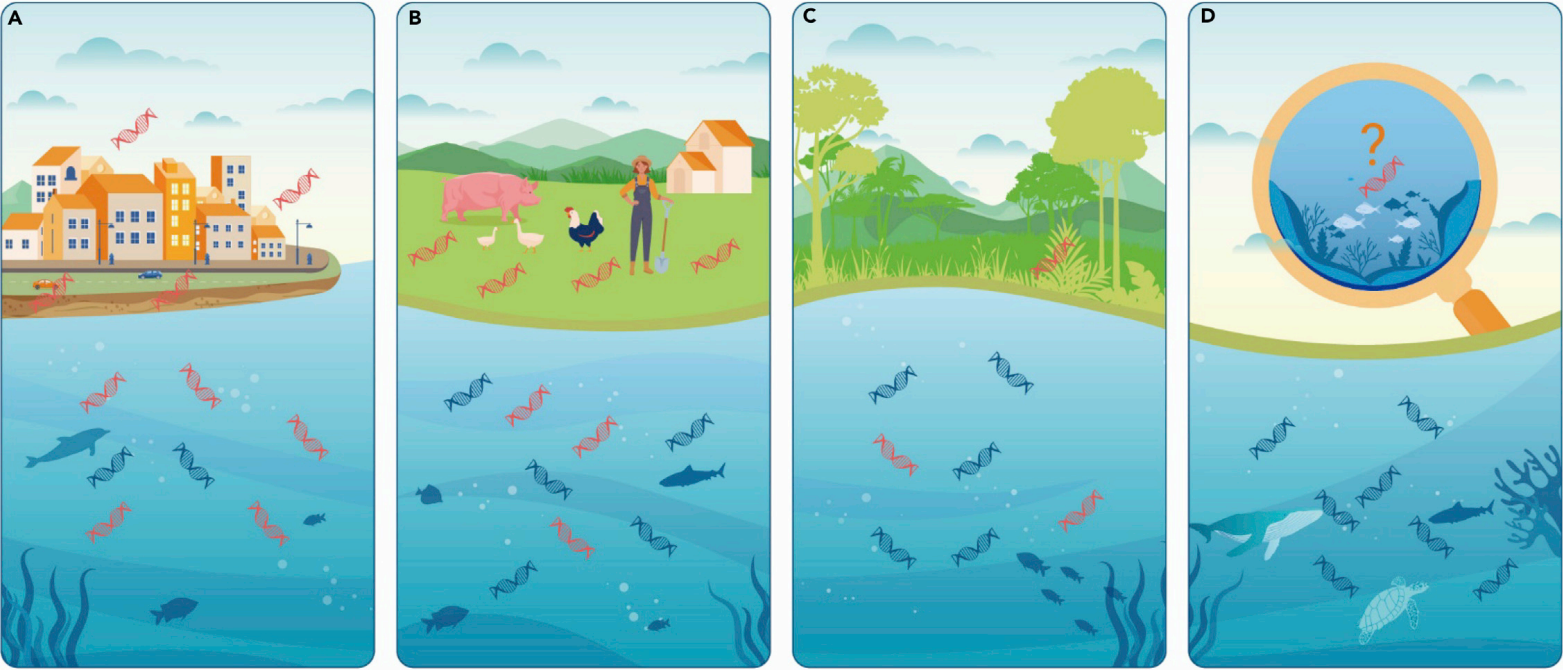
Measuring human and domestic animal eDNA compared with that of resident wildlife may give insight into the human environmental footprint As illustrated in this schematic, relative amounts of human and domestic animal eDNA, depicted in red, compared with wildlife eDNA, depicted in blue, likely differ among urban waterways (A), rural lakes, rivers, and streams (B), remote areas (C), and open ocean (D).

Human action famously transforms the Earth and its biosphere and geosphere.^1^ While most literature consider plowing, burning, debris, and other products and by-products of our activities, direct effects of changing human biomass might merit further study. Humans are now the dominant terrestrial vertebrate, composing about 1/3 of land vertebrate biomass, which translates to 1/10 of total vertebrate biomass.^2^ The other terrestrial 2/3 consists nearly entirely of livestock kept by humans for food. Non-domesticated land vertebrates, from shrews to elephants to swallows, form a mere 3%. Human and domestic animal sequences, commonly detected in environmental DNA (eDNA) metabarcoding studies, are routinely excluded from analysis. Here, we hypothesize that extending eDNA analysis to include human and domesticated animal eDNA (hdaDNA) will provide insight into present-day and potentially historical human impacts on the biosphere (Figure 1). We think the distribution and abundance of hdaDNA are features of the Anthropocene worthy of study.

The growing field of eDNA has shown that the presence and relative abundance of animal species can be assessed by analyzing species-specific DNA in environmental samples.^3^ Most work to date has been done with aquatic eDNA in freshwater and marine habitats. In addition to eDNA of aquatic species, lentic and lotic freshwater systems and near-shore marine waters typically contain eDNA of land animals resident in associated watersheds. Animal eDNA is also present in soil and air. Advantages to eDNA over traditional survey methods include relatively low cost, simplicity of sample collection, performance by a wide variety of personnel, and ability to assess multiple species with a single protocol.

eDNA metabarcoding uses primers that amplify mitochondrial DNA of multiple animal species in a taxonomic group, eg, vertebrates. Metabarcoding amplification products are analyzed by high-throughput sequencing. Each sample generates a library of sequences, called reads. Reads are binned into amplicon sequence variants by a bioinformatic pipeline and are identified to species by matching them to reference databases. In the absence of PCR bias, the share of reads for a given amplicon sequence variant is closely proportional to the number of eDNA copies present in the environmental sample, and, in many systems analyzed so far, the amount of eDNA in the environment is roughly proportional to relative organism abundance. Thus, eDNA reads can provide an index of relative organism abundance. For example, relative fish abundance by eDNA metabarcoding is largely concordant with relative fish abundance as determined by traditional bottom trawl survey.^4^

Vertebrate metabarcoding primers commonly amplify hdaDNA sequences from eDNA samples. As wildlife are usually the species of interest, these sequences are typically filtered out by bioinformatic processing after high-throughput sequencing. Given the large human footprint on the biosphere, we suggest that the distribution of hdaDNA is worthy of study both on its own and compared with wildlife DNA. A critical technical challenge is excluding contamination. For example, negative control libraries prepared from reagent-grade water often contain hdaDNA sequences. Potential sources include operator introduction during water sampling or laboratory processing, PCR and DNA extraction reagents, and cross-contamination by PCR products. It is known that PCR mixes often contain human and domesticated animal DNA. Developing reagents that have no exogenous DNA may be essential. More generally, forensic science techniques designed to avoid DNA contamination of samples should be applicable to eDNA analysis. Absolute quantitation of eDNA copies could enable combining data from different studies, which typically focus on one habitat or region. For instance, it might be possible to combine independent measurements to make a topographic map of human eDNA density. However, metabarcoding tends to amplify all samples to the same number of total reads and so reports relative differences within a sample but not absolute ones. Reads also differ by sequencing depth. Early evidence suggests that spiking metabarcoding PCRs with an internal standard enables quantitation and thus potentially the compilation of data from different studies.^5^ One could also do multiple single-species assays targeting human and domesticated animal species, although that could be cumbersome.

DNA recovered from soil cores and sediments whose layers have been dated could theoretically allow for construction of a historical narrative of the Anthropocene. Similarly, parcels of ocean water from the present, from the recent or distant past frozen in ice or permafrost, or from ancient sources in the slowly circulating ocean conveyor could be assessed for hdaDNA. In these contexts, the importance of excluding contamination is even greater given the very low concentrations of valid eDNA in ancient DNA samples. If this could be solved, then the presence of hdaDNA in a given place and time and its subsequently changing ratio to other taxa has the potential to provide a historical index of human impact during the Anthropocene. Such an index could prove informative in various contexts, such as the interesting question of human roles in the extinction of the mammoth. Historical eDNA studies could also shed light on questions such as the Great Dying in the Americas after 1492, which may have removed more than 90% of the human population, or about 50 million persons.

Atmospheric chemist Paul Crutzen proposed the Anthropocene as a geological epoch characterized by ever-increasing human impact on Earth.^1^ Debate continues over how and when to date the start of the Anthropocene.^1^ Some scholars have argued for the initiation of agriculture more than 12 000 years ago, while others favor the mid-twentieth century when atomic bombs created fallout that spread throughout the atmosphere and oceans. Irrespective of how the Anthropocene is defined, hdaDNA provides a new window for assessing the relative human impacts in ecosystems over space and time. Future studies with titles like “Earth transformed by human action” may include the biogeography of human and domesticated animal eDNA.
